# Application an internet facilitation in a community-based cervical cancer screening project

**DOI:** 10.1186/s12905-023-02733-1

**Published:** 2023-12-01

**Authors:** Hui Du, Xinfeng Qu, Guixiang Wang, Chunlei Guo, Zhaohui Wang, Juan Min, Zhihong Liu, Qicai Hu, Hongxue Luo, Chun Wang, Xia Huang, Yun Chen, Bo Wu, J. L. Belinson, Ruifang Wu

**Affiliations:** 1 Department of Obstetrics and Gynecology, Peking University Shenzhen Hospital, No. 1120, Lianhua Road, Shenzhen, 518036 PR China; 2 Institute of Obstetrics and Gynecology, Shenzhen PKU-HKUST Medical Center, Shenzhen, PR China; 3 Shenzhen Key Laboratory on Technology for Early Diagnosis of Major Gynecologic Diseases, Shenzhen, PR China; 4 Pinshan Renmin Hospital, Shenzhen, PR China; 5 Shenzhen Medical Women’s Association, Shenzhen, PR China; 6 Preventive Oncology International, Inc, Cleveland Heights, OH USA; 7 Women Health Institute, Cleveland Clinic, Cleveland, OH USA

**Keywords:** Cervical cancer screening, Internet-community model, HPV testing, Self-collection

## Abstract

**Objective:**

To evaluate the feasibility of an internet-facilitated community model for cervical cancer screening using self-collected HPV testing as primary screening.

**Method:**

A population-based cervical cancer screening program was conducted in the suburb of Shenzhen, China, from September 2014 to July 2017. Women with 25–60 years of age and no pregnancy were eligible for participation. Participants could register for screening by logging in a website by themselves or with the aids of local community workers. A unique barcode was issued to each applicant upon successful registration. After registration, women could get sampling kits from community screening site/study clinic, collect vaginal samples privately or in group, and provide their sample for Hr-HPV tests on Cobas4800 and SeqHPV assays. Testing reports were checkable through personal account for all participant and phone calls were given to all women positive of Hr-HPV. Participants positive of both or either the 2 assays were identified as the positives. The positives could return the study clinic for triage or search medical care in other clinics. Colposcopy directed or ramdom biopsies were performed on all positives who returned to the study clinics.

**Results:**

A total of 10,792 community women registered for screening, among whom, 10,010 provided their vaginal samples for tests. 99.5% of the participants were confirmed to have correct personal identifiable information and samples, and 98.9% of them got HPV testing results from both or either assays. No adverse event was reported.

**Conclusion:**

When self-collected HPV testing is used as the primary testing, the internet-based data platform facilitates the screening in registration, data collection, and data tracking, and increases the screening coverage. Internet-facilitated community model is promising to cervical cancer control and applicable in regions with variety of resources.

## Background

Cervical cancer is mainly resulted from persistent infection of high-risk human papillomavirus (hrHPV) which contributes to nearly all cases (99%) of the clinically confirmed cervical cancer or precancers [1]. Well-programmed cervical cancer screening has been proven effective in reducing incidence of cervical cancer [1–4]. Nevertheless, the screening coverage remains low in China and other developing countries due to the high medical and financial burdens related to the screening models that highly dependent to medical resources (facilities and personnel).

Most studies on cervical cancer screening have paid much more attention to testing technologies than screening models. However, medical literatures have consensus that key to achieve higher coverage is to reduce the dependence of the screening to medical resources and make it easily accessible for women to participate [5, 6]. In SHENCAST II and several other studies [7–10], we demonstrated that the sensitivity of self-sampling was comparable to provider-sampling using the same type of sampling devices and found that self-sampling significantly facilitated the implementation of population-based screening by decreasing inputs of gynecologists and consumables and saving time for attendants. With self-sampling, we could screen more people with much fewer medical inputs. However, we also need a system including service provision and lab-testing that match the screening effectiveness since self-sampling does not only change the way of sampling. It is important to ensure the easy attendance to the screening and thus we need to further optimize our screening model.

In 2012, we completed the Chinese Cervical Cancer Prevention Study (CHICAPS) [10], in which we developed a community-based participatory model for massive screening using self-sampling and the concepts founded in Community Based Participatory Research (CBPR) Model. The screening was organized by the local community and executed by the community workers (community leaders, CLs). The study showed that communities were able to execute the screening program satisfactorily after a training course of less than 50 munities. Based on the results of CHICAPS, we designed a website for cervical cancer screening and conducted a pilot study on cervical cancer screening through internet services (internet screening model) in 2014, with aim to demonstrate the feasibility of self-registration and sampler/sample delivery [11]. The study demonstrated that a web-based program worked well in providing effective platform for public education, project notification, self-registration for participation, self-sampling instruction, and sampler distribution and sample delivery via general logistic services.

The above-mentioned models facilitate screening attendance, reduce the involvement of medical providers, and allows the healthcare system free from primary screening to focus on evaluation and management of the positives. However, Community-based model is more likely suitable to well-organized population screening in specific timeline and regions and needs a trained team to take screening record, while the internet screening, like online shopping, is only applicable to women who can visit web sites. To make self-sampling-based screening be more applicable, we iterated the website (mcareu.com) we developed earlier and designed a study, in which we integrated internet service into community screening model and apply it in a government sponsored cervical cancer screening project, with a purpose to demonstrate the roles of an public website in a self-HPV testing based population screening program.

## Methods

### Study population

The study was integrated into a population-based cervical cancer screening project that was organized by the local government and applied in Pingshan Xin District, Shenzhen, China. Forty-six (46) community clinics within this district were involved as the screening sites. Women were eligible for participation if they were 25–60 years of age, not pregnant, and with an intact cervix. All women needed to sign up the website to register for participation. Registration for participation need women to fill an online registration form and, if eligible, sign electronic version of informed consent. This study was approved by Ethics Committee in Peking University Shenzhen Hospital under the number G2014-1.

### Study design

The website (www.mcareu.com) was developed by Cervical Cancer Project Team from Peking University Shenzhen Hospital (PUSH) in 2013 and iterated to meet the requirements of the study in 2014. It was designed for public education on cervical cancer prevention, self-sampling instruction, screening registration, result reporting, and further-step guidance for hrHPV-positive women. Assigned community workers from 46 communities in the project area were trained by Gynecologists from PUSH on how to guide the community women to register for participation, to get sample for themselves, and to check results through the website. Then the community workers took missions to notify the communities about the project in their own way, motivate women for participation, and guide women to register for participation via the website. The website was reviewable to all over China, but registration for screening was just opened to the project area we choose to conduct the study. Due to the programmed finance of the local government, we planned to screen 3,000–3,500 women per year.

### Registration for participation and screening delivery

Any person could sign up on the website as the users without limits. Apply for participation needed a woman living in the project area to provide personal identifiable information including her name, ID, phone number, and home address for registration. Any applicant was informed about her serving clinic as the screening site that was near to her living address. With that arrangement, women who were skilled on internet browse were encouraged to complete registration and screening application by themselves, while women who were not skilled on website visits could be aided by the local community workers at the screening sites or their family members at home. A unique barcode would be issued for each eligible applicant upon successful registration. Eligible applicants with a participation barcode would then be called the participants.

The sampling kit used for self-sampling included a conical shaped brush, a specimen processing vial containing PreservCyt liquid, and a graphic sampling guidance. Sampling kits were distributed in the following procedures: They were firstly shipped to all screening sites (the clinic) in batch per the request from each screening site in a full number of participants assigned to the site; then the community workers distributed the sampling kits to the participants.

Before self-sampling, participants could learn self-sampling through referring the graphic guidance provided with the kits or watching the video instruction showing at the screening sites or online of the website. On-site instruction would be provided by the community workers to whomever needed. Completion of screening needed the participants to obtain vaginal samples by themselves and returned the samples to the screening sits. The samples were then sent to PUSH lab for HPV testing.

**Fig. 1:**
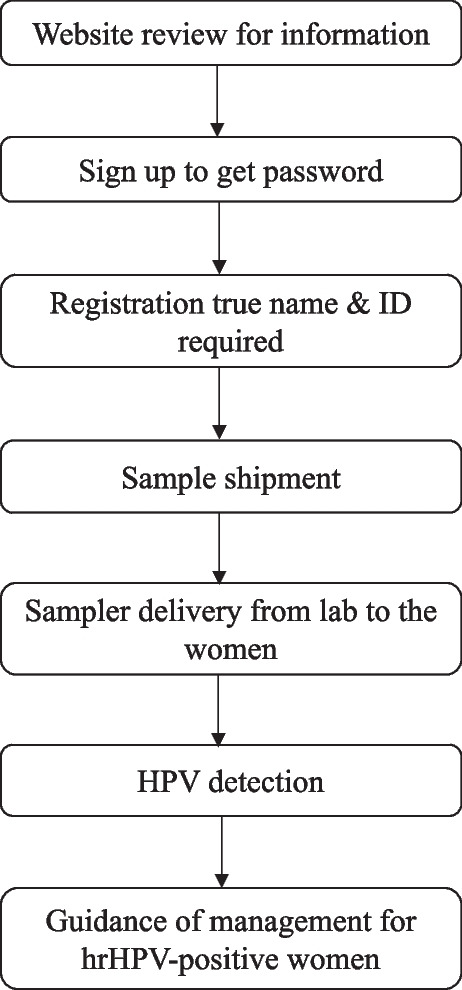
Flow chart for personal screening Shows the flow chart for personal screening. After successful registration, which meant eligible for participation, with basic personal identifiable information including the name, ID, phone number, and home address, the eligible woman was then contacted by a community leader via phone call or text message to confirm her mail-address. A sampling kit with a unique barcode/QC was mailed to her or go to the nearest community health center screening site to pick up a sampler on her own. After getting the kit, she could collect sample for herself and mail the sample to the sample collection address in referring the graphic instruction on the package or following pictures the video instruction playable on the website

**Fig. 2:**
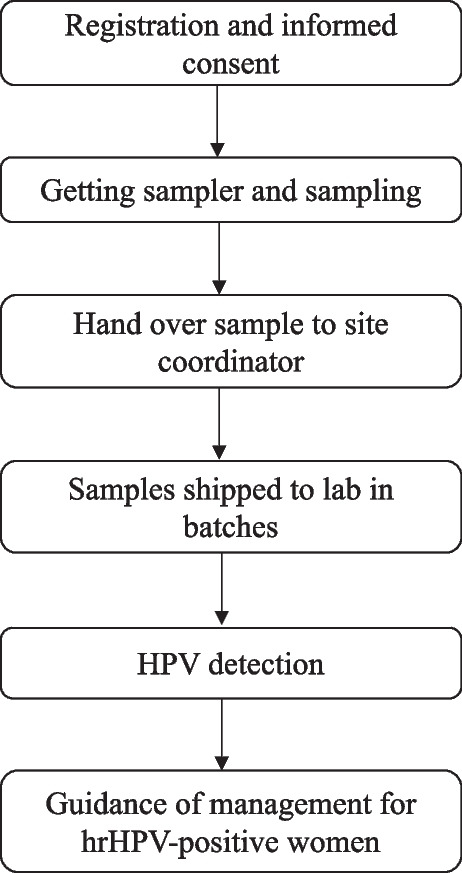
Flow chart for group screening For group screening, a trained medical provider was assigned as a site coordinator at each of the screening sits. The site coordinator organized the screening twice a week at the community site. With consensus of the participants in group, the site coordinator would first sign up for them on the website. After complete registrations for a group of participants, the coordinator distributed the sampling kits to each of the participants one by one. Self-collection was done by the women in a prior prepared sampling room in group (or in the preset cabinets privately) following the graphic or video instructions. The site coordinator would answer all questions from the participants but not get sample for them. At least one small cabinet was set at the site in case that any women would request for a private room for sampling. After sampling, the site coordinator collected the samples and shipped them to the testing lab

When sampling, participants could choose sampling in person or in group per their preference, which mean sampling individually in a private room and at a self-selected time (Fig. 1) or sampling in group in a sampling room in the screening sits with on-site instruction of a community worker (Fig. 2), respectively. The screening events were scheduled twice a week at each of the community sites. During screening events, the community workers could answer all questions from the participants but not collect the sample for the women. In order to ease the participation of those working in factories or companies, temporary sampling rooms were also set in the factory or company’s clinics to complete the screening.

### Sampling and HPV detection

Following the sampling guidance, participants simply inserted the conical-shaped brush into the upper vagina and rotated the brush handle at least three turns, then removed the brush from the vagina and placed it in the vial containing 2.5 ml PreservCyt. All the sample were tested for High-risk types of HPV (hr-HPV) on Cobas4800 (Roche) and SeqHPV (BGI-Shenzhen) assays according to the manufacturer's instructions and within one month.

#### Cobas 4800

The Cobas 4800 (Roche Inc., Pleasanton CA, USA) is a qualitative multiplex HPV testing assay. It provides specific genotyping information for HPV -16 and/or-18 and the pooled result for the other 12 Hr-HPV types (31, 33, 35, 39, 45, 51, 52, 56, 58, 59, 66, and 68). It has been licensed by FDA and CFDA (China Food and Drug Administration). Our previous study has demonstrated that self-collected samples were equal sensitive and specific with physician-collected samples in detection CIN2 + and CIN3 + on Cobas4800 [12, 13].

#### SeqHPV

The SeqHPV testing assay was developed by BGI-Shenzhen, China. It amplifies DNA on multiplex PCR platform and identify HPV genotypes using the next generation sequencing. This assay is advantageous for its high throughput and precise genotypes. SeqHPV was configured to detect 14 Hr-HPV types (16, 18, 31, 33, 35,39, 45, 51, 52, 56, 58, 59, 66, and 68) separately with a through-put of greater than 6,000 samples per unite in a 10-h round at that time. The assay was validated in successive comparisons with Cervista using the specimens and data from SHENCCAST II and then with cobas4800 in a multi-center clinic trial (CHIMUST). It had been licensed by CFDA when used in this study [9, 13].

### Registration data checking and identification of the participation status

All eligible applicants were given a phone call to verify the personal identifiable information on the registration forms and confirm their participation. A case of “data error” would be identified if any registered phone number was not reachable. Participation status was identified as “screened” if a participants visited the screening site for getting a sampling kit and returned her sample, “refusal” for participation if an eligible applicant did not visit the screening site to get a sampling kit after successful registration; or “withdraw” from participation if an eligible applicant got the sampling kit but did not return her sample. Percentages of screened, refusal, and withdraw participants were calculated as the indirect indicators for evaluation of the feasibility of internet facilitated community model.

### Result reporting and management of the positives

Participants who were primarily positive of Hr-HPV on both or either SeqHPV and/or Cobas4800 were defined as the positives. The primary Hr-HPV testing results were reported online, together with the recommendations for further management. Participants could access their personal results by signning on the website with the username or ID and the passwords. Text message validation would be needed if the password was forgotten. A text message notifying the availability of the testing report was sent to the registered mobile phone number of all the participants. Following the text message notification were phone calls by the research team to all the positives with aim to inform them the positive results, explain the results, answer their questions, release their anxiousness, instruct them to search medical cares, and help them to make appointments for triages in the study clinics. All the positives were encouraged to return to the study clinics for triages, where triage clinic were provided by gynecologists from PUSH every three months. The participants were also informed that they had right to search medical care for their positive results in qualified medical facilities other than the study clinics. To the positives who returned to the study clinics, colposcopy and biopsy were conducted following a protocol of colposcopy-directed and random multi-biopsies. Histological diagnostics were conducted by pathologists from PUSH.

## Results

### Characteristics of the participants

This study was conducted during September 2014 to April 2017. Twelve thousand six hundred and ninety-nine (12,699) people signed up the website, among them 10,792 successfully registered for the screening (the participants) and contacted for provision of sampling kit. Of the participants, 10,010 (92.8%) provided their self-collected samples and were identified as the screened participants. Of the 782 participants who did not provide samples, 496 (63.4%) did not visit the screening sites to get sampling kits and was identified as “refusal” for participation; while 286 (36.6%) got the samplers but did not provide samples and were identified as “withdraw” from participation. Nine thousand nine hundred and thirty-eight (9,938, 99.3%) participants were confirmed with provision of correct personal identifiable data, with a data error rate of 0.7%.

Included in the screened participants were 62.8% (6,286/10,010) of community residents, 12.8% (1,281/10.010) of medical providers, 10.8% (1,081/10,010) of factory workers, 7.8% (781/10,010) of company clerks, and 6.4% (641/10,010) of government officers. The mean age of the screened participants was 35.9 years (± 7.48) (Table 1).

**Table 1:** Participant categorization

Participant classification	Age(y)	n	%
Community Residents	36.4 ± 7.40	6,294	62.8%
Medical providers	38.5 ± 7.80	1,277	12.8%
Plant workers	33.6 ± 6.38	1,022	10.2%
Clerks from company	29.7 ± 7.47	778	7.8%
Government officers	36.7 ± 7.44	639	6.4%
Total	35.9 ± 7.48	10,010	100%

Of the screened participants, 26.6%(2,666/10,010) collected their samples privately (the personal screening group) and 73.4%(7,344 /10,010) got their samples in group at the local screening sites (the group screening group). The mean-ages of the personal screening group and the group screening groups were 39.9 ± 7.94 years and 34.4 ± 6.73 years, respectively. And the ratio of women with ≥ 50 years of age were 9.9% (265/2,666) and 2.1%(156/7,344) in the personal and group screening groups, respectively (Table 2). Most of the participants from factories or companies were young women and they were screened in group, thus the average age of group screening group was younger than those in personal screening group. Data errors were identified in 0.64% (17/2,666) and 0.76% (56/7,344) of the participants in personal- and group-screening groups, respectively, with no significant deference (Table 2).

**Table 2:** The screening women age, errors and testing failures

Screening choice	Mean age (yrs)	> 50 yrs	Information error
Personal screening	39.9 ± 7.94	9.9% (265/2666)	0.64% (17/2666)
Group screening	34.4 ± 6.73	2.1% (156/7344)	0.76% (56/7344)
Total	35.9 ± 7.48	4.2%(421/10010)	0.73% (73/10011)

### Primary testing results

A total of 1,135 (11.34%) of the participants were tested positive of hrHPV on Cobas 4800 and/or SeqHPV assays (Table 3). Overall, 10.51% of participants were tested Hr-HPV positive on Cobas4800, and 7.56% were positive on SeqHPV (Table 3). Testing failures were reported to be 1.20% (120/10010) on Cobas4800 and 1.05% (105/10010) on SeqHPV, with no significant difference between the two assays. It is addressed that only 12 (0.12%) participants got testing failure on both two assays who were identified as sampling failure, suggesting that most testing failure is not related to self-collected samples. We contacted all those 12 participants with no HPV results for re-sampling. Unfortunately, 11 of them refused to return and one got negative result for her re-collected sample.

**Table 3:** Primary hrHPV testing results

Screening test	Positives	% (positive)	Invalid	% (Invalid)
Cobas	1052	10.51	120	1.20
SeqHPV	757	7.56	105	1.05

### Call back for management of triage of the hrHPV-positive women

Reports for primary Hr-HPV testing were released on to the website for private review with the personal account control. A text message was sent to the screened participants after the reports were reviewable online to notify them the report-checkability only. Exactly after the text messages were sent out, the community workers would contact the 1,135 participants who were tested positive of Hr-HPV (the positives) via phone call. However, only 1,045 positives had been contacted, with 7.93% (90/1135) of loss-follow-up for variety of reasons. Since the participants were allowed to search medical cares in their preferable medical facilities, we eventually had 65.9% (689/1,045) of the approachable positives return back for triage (Table 4). All CIN2 + patients were referred for treatment in PUSH or other qualified hospitals following standard clinic procedures.

**Table 4:** Positive management

Any hrHPV positive (Cobas or SeqHPV)	1135	11.34% (1135/10011)
The return rate for colposcopy	689	60.70% (689/1135)
Follow-up loss	446	39.30% (446/1135)

## Discussion

Programed screening is a working way to prevent cervical cancer that is threatening women’s health worldwide. World Health Organization (WHO) recommends provision of programed screening as the effective strategy to prevent cervical cancer. The hospital centralized screening is easy to conduct as it just needs the patients to visit hospital regularly and the doctors to follow the recognized guidelines for the clinical patient care. Under this way, we will eventually get the right answer for a specific woman. However, such screening manner has been demonstrated to contribute little for cervical cancer control in populations, especially in medically underserved regions. When we are projecting to goal successful cervical cancer prevention for a country or city with large population, the screening program should be characterized as: (1) high in coverage, (2) simple for implementation, (3) cost affordable, and (4) highly sensitive.

We have known that HPV testing for self-collected samples is equally accurate to physician-collected ones. And self-samples have been proven to be efficient and convenient for screening by means of enlarging screening coverage and enabling community screening, particularly in low-resource areas [7–9, 14]. However, we had some problems that were not about technology, but about the screening organization.

Cervical cancer screening programs were usually organized by the local authorities with the assistance from gynecologists and implemented within medical facilities. In addition, women had to visit the local hospitals for screening in a specified duration of time. This caused high burden for both the organizers and the attendants. By contrast, it should be much more convenient for women if the screening program could be organized within the local communities where they live in. During 2011–2012, using a community based participatory research model, we first designed and studied a community screening system, in which short-term training coursed on self-sampling-based project procedures were provided to the local community staff, who then successfully organized self-sampling-based screening for 8,382 women in rural regions in Guangdong Province, China. However, we also realized that community screening model is only suitable to screen women who could be well organized in specific time and region and such screening still need personnel, although not medical providers, from the community to record screening information, which is a challenge to community workers who are not medically backgrounded. A date platform is needed to facilitate the community screening to easy the data recording and to guarantee the quality of the data.

With the above consideration in mind and to make the community-based screening model be feasible in different regions, we designed and developed a public data platform that was based on internet system to facilitate the community screening. Given the wide coverage of internet in our daily life, the website can promote dissemination of cervical cancer prevention knowledge, improve the convenience for participation, and facilitate management of screening. With the data platform on the website, screening organizers, doctors, and researchers can accumulate and keep long-term screening data, which is important for analyzing the screening coverage and strategizing prevention programs. In a pilot study on internet screening we conducted in 2013, 1,000 women completed primary screening via the same website (mcareu.com). Procedures they went through on the website included application and registration for screening, getting sampling kits, sending samples to the lab, and checking screening results online [11]. This pilot study made us be confident on the feasibility of the internet-based screening model because the error of the data input by the screening women themselves was rated zero percent. In addition, combination of internet facilitation and community screening model can reduce the medical burden when primary HPV testing can be organized and managed by local community staff without involvement of medical staff.

Results from this study showed no significant differences between the personal and group screening groups in testing failures and personal information errors, suggesting that, with aids from the community worker, women unskilled for internet visits could finish online application and screening as well as the skilled women. Private screening is advantaged for its fair privacy but disadvantaged for the high per-sample cost for sampler/sample shipments, comparatively, group screening enables dramatic decrease of the shipment cost.

Positive management rate was not objected to be evaluated in our study. Thus, we gave the hrHPV-positive women free choice for management at the appointed site by PUSH team for free of charge or searching for payable service from any other hospital. However, in noticing that 60.7% of the positives returned for free positive management by the PUSH team in 3 months interval, we also analyzed the potential reasons which suggests that 1) publicly recognized high-level medical cares was more preferrable to public women, 2) the cervical cancer screening program should make primary screening, positive triage, and precancer treatment as the inseparable parts, and 3) free of charge for all the program procedures may be a working way to get high rate of treatment for precancers, the core step of cancer control. To include management of hrHPV-positive women in the screening programs, especially in those to be implemented in remote areas, we need to make the management be applicable in primary medical facilities as closer to the communities as possible by training more primary level of medical providers and verifying variety of protocols for triage that suitable for those providers. Encouragingly, the screening model we developed was supported later by full-coverage screening for Covid-19 in the pandemic stage, which adopted similar internet platform to identify people, collect samples, release reports, and keep records for checking.

Another problematic issue is the property of liquid-based sample storage to self-sampling, which caused overflow and was hardly transported for a long-distance in our study. As FTA-card based solid medium had been demonstrated to work well for DNA transportation for HPV testing, it can play important roles in self-sampling based population screening projects [15, 16].

In our study, we enrolled participants who were aged 25–60 of years. The reason for including women younger than 30 is that the study was integrated into a government organized cervical cancer screening project which tended to cover women at age of 25–60 years.

In conclusion, internet & community-based screening model holds the potentiality to be widely applied in regions with different levels of medical and economic resources. With this model, community staff can promote and manage cervical cancer screening continuously. With facilitation of the internet system, community-based screening using self-sampling can achieve greater coverage in a shorter period. This conclusion was further evidenced by Qu at els., who shared a population screening project conduced in Xinxiang, Henan, China, using internet facilitated community screening model. In the project, the internet facilitation enables 272,004 women registered in 33 days, and 188,096 be screened in 29 days [17].

## Data Availability

The datasets used and/or analyzed during the current study available from the corresponding author on reasonable request.

## References

[1] Mayrand MH, Duarte-Franco E, Rodrigues I, Walter SD, Hanley J, Ferenczy A, Ratnam S, Coutlee F, Franco EL, cervical cancer screening trial study group (2007). Human Papillomavirus DNA versus Papanicolaou screening tests for cervical cancer. N Engl J Med.

[2] Naucler P, Ryd W, Tornberg S, Strand A, Wadell G, Elfgren K, Radberg T, Strander B, Johansson B, Forslund O, Hansson BG, Rylander E, Dillner J (2007). Human papillomavirus and Papanicolaou tests to screen for cervical cancer. N Engl J Med.

[3] Ronco G, Dillner J, Elfström KM, Tunesi S, Snijders PJ, Arbyn M, Kitchener H, Segnan N, Gilham C, Giorgi-Rossi P, Berkhof J, Peto J, Meijer CJ, International HPV screening working group (2014). Efficacy of HPV-based screening for € prevention of invasive cervical cancer: follow-up of four European randomised controlled trials. Lancet..

[4] Wright TC, Stoler MH, Behrens CM, Sharma A, Zhang G, Wright TL (2015). Primary cervical cancer screening with human papillomavirus: end of study results from the ATHENA study using HPV as the first-line screening test. Gynecol Oncol.

[5] Wright TC, Denny L, Kuhn L, Pollack A, Lorincz A (2000). HPV DNA testing of self-collected vaginal samples compared with cytologic screening to detect cervical cancer. J Am Med Assoc.

[6] Gravitt PE, Rositch AF (2014). HPV self-testing and cervical cancer screening coverage. Lancet Oncol.

[7] Belinson JL, Qiao YL, Pretorius RG, Zhang WH, Elson P, Li L, Pan QJ, Fischer C, Lorincz A, Zahniser D, Shanxi Province Cervical Cancer Screening Study (2001). A cross-sectional comparative trial of multiple techniques to detect cervical intraepithelial neoplasia. Gynecol Oncol.

[8] Belinson JL, Du H, Yang B (2012). Improved sensitivity of vaginal self-collection and high-risk human papillomavirus testing. Int J Cancer.

[9] Yi X, Zou J, Xu J, Liu T, Liu T, Hua S, Xi F, Nie X, Ye L, Luo Y, Xu L, Du H, Wu R, Yang L, Liu R, Yang B, Wang J, Belinson JL (2014). Development and validation of a new HPV genotyping assay based on next-generation sequencing. Am J Clin Pathol.

[10] Belinson JL, Wang G, Qu X, Du H, Shen J, Xu J, Zhong L, Yi J, Yi X, Wu R (2014). The development and evaluation of a community based model for cervical cancer screening based on self-sampling. Gynecol Oncol.

[11] Wu R, Qu X, Du H, Liu Z, Hu Q, Wang C, Zhang L, Zhao J, Wang G, Belinson JL (2016). A pilot study to evaluate an internet-based cervical cancer screening model based on self-sampling. Health.

[12] Isidean SD, Coutlée F, Franco EL (2014). cobas 4800 HPV Test, a real-time polymerase chain reaction assay for the detection of human papillomavirus in cervical specimens. Expert Rev Mol Diagn.

[13] Du H, Wang G, Zhang W, Wang C, Hu Q, Kang H, Li Y, Wu R. To evaluate the sensitivity of Cobas 4800 HPV test and Seq HPV Test in detecting vaginal self-samples in screening. In: 29th International Papillomavirus Conference and Public Health & Clinical Workshops, Seattle. 2014. p. 97.

[14] Arbyn M, Verdoodt F, Snijders PJ, Verhoef VM, Suonio E, Dillner L, Minozzi S, Bellisario C, Banzi R, Zhao FH, Hillemanns P, Anttila A (2014). Accuracy of human papillomavirus testing on self-collected versus clinician-collected samples: a meta-analysis. Lancet Oncol.

[15] Luo H, Du H, Maurer K, Belinson JL, Wang G, Liu Z, Zhang L, Zhou Y, Wang C, Tang J, Qu X, Wu R (2016). An evaluation of the Cobas4800 HPV test on cervico-vaginal specimens in liquid versus solid transport media. PLoS One.

[16] Maurer K, Luo H, Shen Z, Wang G, Du H, Wang C, Liu X, Wang X, Qu X, Wu R, Belinson J (2016). Evaluation of a new solid media specimen transport card for high risk HPV detection and cervical cancer prevention. J Clin Virol.

[17] Du Y, Zhang L, Wu R, Qu X. Development and application of an internet-facilitated community-based participatory model for cervical cancer screening. Atlanta: 2019 ASCCP Annual Scientific Meeting on Anogenital and HPV-Related Diseases; 2019.

